# Impaired Pten Expression in Human Malignant Peripheral Nerve Sheath Tumours

**DOI:** 10.1371/journal.pone.0047595

**Published:** 2012-11-06

**Authors:** Maren Bradtmöller, Christian Hartmann, Jan Zietsch, Sebastian Jäschke, Victor-F Mautner, Andreas Kurtz, Su-Jin Park, Michael Baier, Anja Harder, David Reuss, Andreas von Deimling, Frank L. Heppner, Nikola Holtkamp

**Affiliations:** 1 Department of Neuropathology, Charité - Universitätsmedizin Berlin, Berlin, Germany; 2 Department of Neuropathology, Institute of Pathology, Ruprecht-Karls-University Heidelberg, and Clinical Cooperation Unit Neuropathology, German Cancer Research Center, Heidelberg, Germany; 3 Department of Maxillofacial Surgery, University Hospital Eppendorf, Hamburg, Germany; 4 Berlin-Brandenburg Center for Regenerative Therapies, Charité - Universitätsmedizin Berlin, Berlin, Germany; 5 College of Veterinary Medicine, Seoul National University, Seoul, Republic of Korea; 6 Project Neurodegenerative Diseases, Robert-Koch-Institute, Berlin, Germany; 7 Institute of Neuropathology, University Hospital Münster, Münster, Germany; Mayo Clinic, United States of America

## Abstract

Malignant peripheral nerve sheath tumours (MPNST) are aggressive sarcomas that develop in about 10% of patients with the genetic disease neurofibromatosis type 1 (NF1). Molecular alterations contributing to MPNST formation have only partially been resolved. Here we examined the role of Pten, a key regulator of the Pi3k/Akt/mTOR pathway, in human MPNST and benign neurofibromas. Immunohistochemistry showed that Pten expression was significantly lower in MPNST (n = 16) than in neurofibromas (n = 16) and normal nervous tissue. To elucidate potential mechanisms for Pten down-regulation or Akt/mTOR activation in MPNST we performed further experiments. Mutation analysis revealed absence of somatic mutations in *PTEN* (n = 31) and *PIK3CA* (n = 38). However, we found frequent *PTEN* promotor methylation in primary MPNST (11/26) and MPNST cell lines (7/8) but not in benign nerve sheath tumours. *PTEN* methylation was significantly associated with early metastasis. Moreover, we detected an inverse correlation of Pten-regulating miR-21 and Pten protein levels in MPNST cell lines. The examination of *NF1*−/− and *NF1*+/+Schwann cells and fibroblasts showed that Pten expression is not regulated by *NF1*. To determine the significance of Pten status for treatment with the mTOR inhibitor rapamycin we treated 5 MPNST cell lines with rapamycin. All cell lines were sensitive to rapamycin without a significant correlation to Pten levels. When rapamycin was combined with simvastatin a synergistic anti-proliferative effect was achieved. Taken together we show frequent loss/reduction of Pten expression in MPNST and provide evidence for the involvement of multiple Pten regulating mechanisms.

## Introduction

Malignant peripheral nerve sheath tumours (MPNST) are aggressive soft tissue sarcomas that develop with an incidence of 1∶100.000. Although rare in the general population MPNST develop frequently in patients with the genetic disorder neurofibromatosis type 1 (NF1), which occurs with an incidence of 1∶3500 [1]. The life-time risk of developing MPNST is estimated to be 8–13% [2] for NF1 patients, who account for about 50% of all MPNST. MPNST generally arise from plexiform neurofibromas (pNF) in NF1 patients and constitute the major cause for reduced life expectancy with only 21% of patients surviving longer than 5 years after diagnosis. Loss of the tumour suppressor gene (TSG) *NF1* is only a first step in tumourigenesis. During the course of malignant progression, further alterations are acquired in TSG and oncogenes like *TP53*, *CDKN2A*, *EGFR*, and *PDGFRA* [3], [4], [5], [6], [7]. Although sporadic and NF1-associated MPNST share many similarities in their molecular pathogenesis, Ras mutations are linked to sporadic MPNST whereas *PTEN* monosomy segregates with NF1-associated cases [8]. The currently dim treatment options for MPNST patients may be improved by a better knowledge on molecular alterations, which could lead to novel strategies of targeted therapy. Neurofibromin, the *NF1* gene product, is a negative regulator of the Ras oncoprotein. Moreover, it was shown that the Akt/mTOR (mammalian Target of Rapamycin) pathway is activated in *NF1* deficient cells [9]. This pathway is attractive for targeted therapy since different mTOR inhibitors are already approved for clinical application. Recently we found allelic loss of *PTEN* (Phosphatase and tensin homologue deleted from chromosome 10) in 58% MPNST [7]. Pten protein is a major regulator of the Pi3k/Akt/mTOR pathway. Loss or down-regulation of Pten expression leads to the activation of this pathway and thus promotes malignant progression. *PTEN* is the second most frequently altered TSG and inactivated in a variety of tumour entities including glioblastoma, prostate cancer and melanoma. Pten has lipid phosphatase activity and dephosphorylates phosphatidylinositol-(3,4,5)-triphosphate (PIP3) to phosphatidylinositol-(4,5)-bisphosphate (PIP2). Thereby it antagonizes the activity of the phosphatidylinositol-3-kinase (Pi3k) which converts PIP2 to PIP3. Via this mechanism Pten controls the Akt/mTor pathway, which promotes multiple functions, including cell growth and survival, proliferation, apoptosis, invasion, migration and angiogenesis.

Recently, a transgenic mouse model provided evidence for an important role of Pten in development of benign and malignant nerve sheath tumours [10]. The authors demonstrated that in addition to a constitutively active K-Ras mutant a reduced *Pten* dosage was necessary for tumour formation. Deletion of both *Pten* alleles was observed in malignant but not in benign nerve sheath tumours. This study points towards a crucial role of Pten in nerve sheath tumour formation, however, the employed mouse model does not reflect the genetic nature of NF1 patients and the question why mice haploinsufficient for *Pten* and *Nf1* completely lacked tumour development remains unsolved.

Here we determined the frequency of Pten alterations in human MPNST and neurofibromas and examined underlying mechanisms.

## Materials and Methods

### Tumour Tissue, DNA and RNA Extraction

Paraffin embedded and frozen tumour and nerve samples were collected in the following German hospitals: University Hospital Eppendorf (Hamburg), Otto-von-Guericke-University (Magdeburg), Robert-Rössle-Hospital (Berlin), and Charité – Universitätsmedizin Berlin. Following initial diagnosis in local neuropathologies, all tumour samples were reviewed by the same experienced pathologist (AvD). Tumour sections were examined histologically prior to extraction of nucleic acids and proteins. DNA and RNA from frozen tumours (6 MPNST and 9 neurofibromas), all cell lines and cell cultures were extracted with Trizol reagent (Invitrogen, Karlsruhe, Germany). RNA integrity was analysed with a Bioanalyzer from Agilent (Böblingen, Germany). Samples with an RNA integrity number (RIN)<7 were excluded. RIN of cell lines was >9. DNA extraction from paraffin embedded material was carried out according to the QIAamp DNA Mini Kit protocol (Qiagen, Hilden, Germany). The investigations were carried out with the informed consent of the patients.

### Immunohistochemistry and Scoring

Immunohistochemistry on paraffin embedded slices was performed with the BenchmarkTM system from Ventana (Strasbourg, France). Pten antibody (A2B1, dilution 1∶80) was obtained from Santa Cruz Biotechnology (Heidelberg, Germany). Visualization was performed with diaminobenzidine. Negative controls without primary antibodies were carried out. Scoring was performed according to the percentage of positive cells: <5% was classified as negative (−), 6–100% was classified as positive. 6–30% of positive cells were scored with +, 31–60% with ++, >60% with +++. A blinded repeated test produced similar results.

Immunofluorescence double staining was performed manually. Antigen retrieval was achieved by heating. Pten (A2B1, dilution 1∶80) S100 and neurofilament from DakoCytomation GmbH (Hamburg, Germany), dilution 1∶1000, antibodies were used. For visualization we utilized 1∶100 dilutions of Cy3- and Alexa Fluor 488-conjugated antibodies. Nuclei were counterstained with DAPI. Normal skin tissue served as positive control. Slices were photographed with the confocal laser mikroscope LSM5 Exciter from Zeiss (Jena, Germany).

### *PTEN* and *PIK3CA* Mutation Analysis

The nine coding exons of *PTEN* were sequenced bidirectionally with nine primer pairs labelled either with M13-forward and –reverse sequences conferring PCR products between 160 and 364 bp. PCR was performed in a volume of 15 µl with 20 ng of DNA, GoTaq DNA Polymerase (Promega, Mannheim, Germany) applying 35 cycles. Primer sequences are available on request. For sequencing the BigDye Terminator v3.1 Sequencing Kit (Applied Biosystems, Darmstadt, Germany) was utilized. For *PIK3CA* analysis a set of five primer pairs was employed, covering exon 1, 9 and 20. The primer sets [11], PCR and single strand confirmation polymorphism (SSCP) have been described before [12]. For enhanced sensitivity all PCR products were separated on 8% and 14% acrylamide gels.

### *PTEN* Promotor Methylation Analysis

Promotor methylation was done as previously described [13] and performed by Varionostic GmbH (Ulm, Germany). Briefly, DNA was bisulfite-treated followed by quantitative positional methylation analysis (pyrosequencing). The methylation analysis was restricted to CpG island 3 which contains 5 CpG sites. A methylation of<8% of DNA was regarded as unmethylated. The assay does not detect methylation of the *PTEN* pseudogene *PTENP1*.

### Expression Analysis by Real Time RT-PCR

Reverse transcription and DNA digestion of 1 µg RNA was achieved with the Quantitect reverse transcription kit (Qiagen, Hilden, Germany). Subsequent PCR reactions were performed with Taqman Mastermix FAST and gene specific MGB probes. PCRs were performed in triplicates in a volume of 20 µl containing cDNA equivalents of 10 ng RNA. PCR reagents, probes and the 7900 HT fast real time PCR system were from Applied Biosystems (Darmstadt, Germany). Data were accepted as valid if the standard deviation of Ct values (threshold cycles) of triplicate reactions was <0.5 cycles. *ACTB* and *RPS3* were employed as endogenous controls.

Expression of miR-21 and miR-217 was analysed with the Taqman microRNA reverse transcription kit and subsequent amplification with Taqman Universal PCR master mix and Taqman microRNA assays (all from Applied Biosystems). RNU44 was used as endogenous control.

### Western Blots

Subconfluent cell cultures were lysed in ice cold lysis buffer (1% Triton X100, 100 mM NaCl, 50 mM Tris-HCl pH 7.5, 5 mM EDTA) containing protease and phosphatase inhibitor cocktail. For lysates of solid tumours 1–3 g of tissue was homogenized in lysis buffer. After heat denaturation approximately 40 µg per sample was loaded on to 12% acrylamide gels. For comparability of different gels cell line T265 was run on every gel as an internal standard. After blotting membranes were stained with Ponceau S to verify protein transfer. Membranes were blocked and incubated overnight at 4°C with primary antibodies. The p-p70S6 kinase antibody (detects also the p-p85S6 isoform, dilution 1∶2000 (R&D Systems, Wiesbaden, Germany) and Pten antibodies (either A2B1 from Santa Cruz Biotechnology or Pten antibody from Zytomed, both with1∶200 dilutions) were used. The β-actin antibody (AC-15, dilution 1∶6.000) was from Sigma (Munich, Germany). After washing membranes were incubated for 1 h with second horseradish conjugated antibodies. Visualization was performed with ECL or advanced ECL (Amersham Biosciences, Freiburg, Germany).

### Cell Culture Assays

All cell lines were maintained in DMEM Glutamax-I (1000 mg/L glucose) with 10% FCS and 5 µg/mL gentamycin from Invitrogen (Karlsruhe, Germany). MPNST cell lines S462, S520, 1507.2, S805 were established and provided by V. F. Mautner, University Hospital Eppendorf, Germany [14]. MPNST cell line STS26T was provided by G. H. De Vries (Hines VA Hospital, Illinois, USA) [15]. MPNST cell line ST88-14 was provided by J. DeClue (NIH, Bethesda, USA) [16]. NFS-1, low passage culture 31002 and dermal fibroblasts have been described elsewhere [7], [17]. Mouse embryonal fibroblasts from *Nf1* wildtype and *Nf1−/−* mice were prepared and genotyped by D. Kaufmann (Institute of Human Genetics, Ulm, Germany) [18]. With the exception of MPNST cells 31002 and STS26T, which were obtained from sporadic MPNST, all other MPNST lines were from NF1 associated MPNST. Neurofibroma derived Schwann cells were cultured on laminin coated dishes and cultured in DMEM supplemented with 10% FCS (Invitrogen), 2.5 µg/ml insulin (Sigma, München, Germany), 0.5 mM IBMX (Serva, Heidelberg, Germany), and 10 nM heregulin (Peprotech, Hamburg, Germany). 0.5 µM forskolin from Sigma was used for normal Schwann cells (Sciencell, Carlsbad, CA) but not for neurofibroma derived *NF1*−/− Schwann cells since these cells are selectively expanded when forskolin is omitted [19]. For comparative analysis Schwann cells were set to the same medium conditions (DMEM 0.5% FCS) for at least 72 h before harvest. When proliferation assays were performed cell lines were maintained in DMEM containing 5% serum. 3×103 cells were seeded in 300 µL medium into 24 well plates and allowed to adhere over night. Drugs were added in 100 µL to obtain the indicated concentrations. Negative controls contained vehicle only. Cell proliferation was evaluated on day 4 post-treatment with the CellTiter 96 AQueous One Solution Cell Proliferation Assay (Promega, Mannheim, Germany). The experiments were performed in quadruplicate and repeated at least three times. Rapamycin and simvastatin were purchased from Calbiochem (Darmstadt, Germany). Fractional product concept was used to determine whether drug combinations yielded additive or synergistic effects.

### Determination of Phospho mTOR/total mTOR and VEGF

Phosphorylated mTOR and total mTOR were simultaneously determined with the MSD 96-well Multi-spot Phospho (Ser2448)/total mTOR assay from Meso Scale discovery (Gaithersburg, MD). VEGF concentration was measured in with the Multi-array 96-well plate from Meso Scale discovery. Optimal protein concentration was between 10–20 µg in 25 µl as determined by serial dilution. The assay was performed according to the manufacturer’s recommendation.

### Statistical Methods

SPSS version 12.0 was used for statistical analysis. Survival rates were determined using the Kaplan–Meier method and the log rank test. Association of parameters was assessed with Pearson correlation and Fisher exact test. A p-value of <0.05 was considered as significant.

## Results

### Pten Expression in Nerve Sheath Tumours and Nervous Tissue

**Figure 1:**
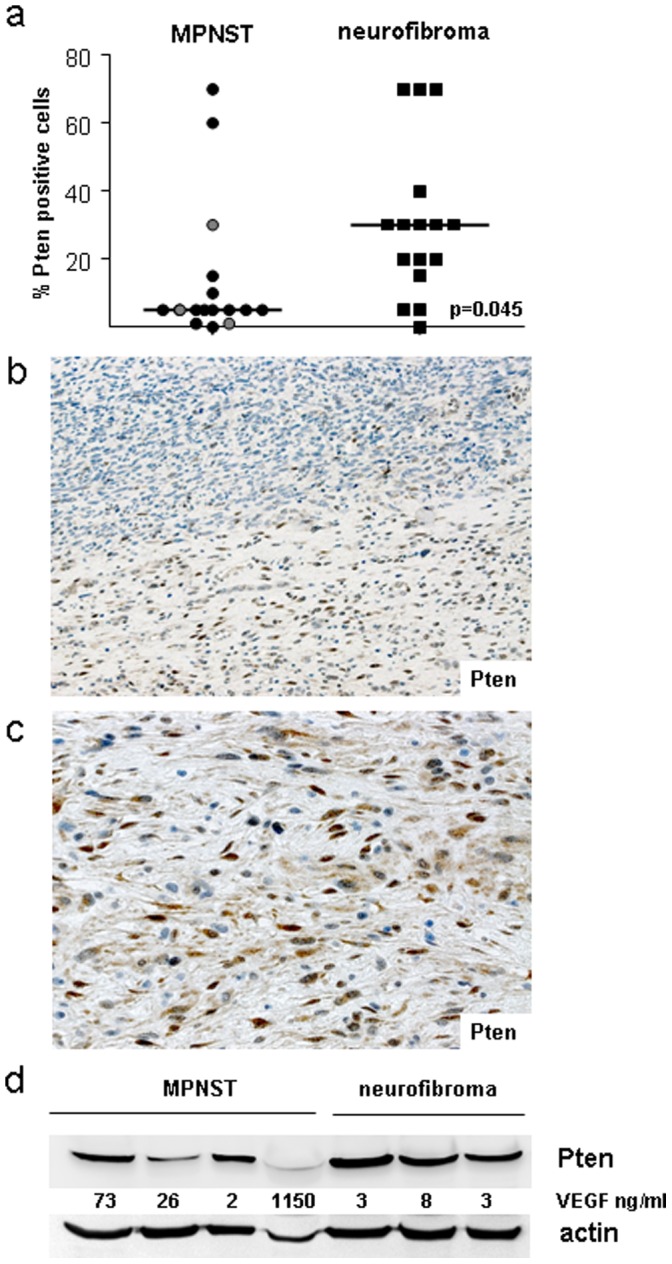
Pten expression in MPNST and neurofibroma. a) Proportion of Pten-positive tumour cells as determined by immunohistochemistry. Each dot represents one tumour. Sporadic MPNST are depicted in grey b) MPNST 29250 with a transition to pNF. Note stronger Pten expression in the pNF (lower part). c) Higher magnification of pNF. Original magnification 200x and 400x. d) Western blot of MPNST and neurofibromas. VEGF concentrations in tumour lysates are indicated.

Pten immunohistochemistry shows a significantly (p = 0.045, Pearson correlation) reduced Pten expression in MPNST (n = 16, 3 sporadic and 13 NF1-associated MPNST) as compared to neurofibromas (n = 16) (Figure 1a–c). Median proportion of Pten positive cells was 30% in neurofibromas and 5% in MPNST. In line with these data, three tumours with transition from pNF to MPNST showed lower Pten expression in the malignant part. An example is shown in Figure 1b, with 5% Pten positive cells in the MPNST versus 70% positive cells in the pNF part. Figure 1c shows Pten expression pattern in the pNF with typical spindle shaped cell morphology. Pten staining was visible in the cytoplasm and in the nucleus. Given that nuclear Pten has been associated with more differentiated, resting cells we compared its subcellular distribution in our nerve sheath tumour samples. The ratio of nuclear Pten was higher in neurofibromas as compared to MPNST (mean of 50% versus 30% as shown in Figure S4).

Western blot analysis shows reduced Pten expression particularly in 2 of 4 MPNST (Figure 1d). However, in tumour lysates utilized for Western blot, non-tumourous cell types like endothelial cells contribute to Pten signals. Production of vascular endothelial growth factor (VEGF) increases upon activation of the Akt/mTOR pathway. Since phosphorylated proteins were difficult to detect in lysates from primary tumours, we determined VEGF levels and found stronger expression in MPNST than in neurofibromas (Figure 1d).

**Figure 2:**
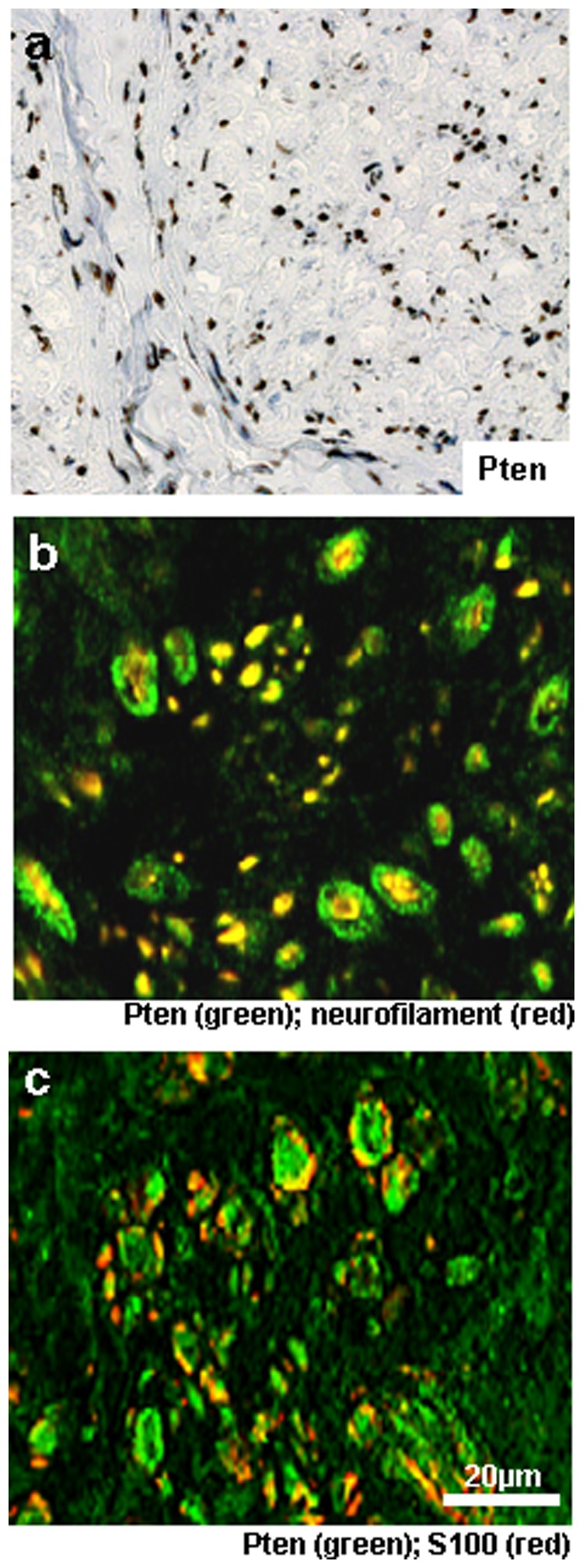
Pten expression in nerve tissue (a-c) and Schwann cell cultures (d). a) Standard DAB staining. b) Double staining for Pten (green) and neurofilament (red). c) Double staining for Pten (green) and S100 (red). Original magnification 400x.

In order to compare Pten expression levels of healthy nerve tissue with nerve sheath tumours we examined three nerves without diagnostic findings (Figure 2). Because all 3 samples were highly positive we aimed to find out the exact cell type expressing Pten in nervous tissue. Immunoflourescence double staining revealed expression of Pten in neurofilament-positive axons as well as in S100-positive Schwann cells (Figure 2b & c). The cross section in Figure 2b shows nerve fibers with a central axon, double positive for neurofilament and Pten, ensheathed by Pten positive Schwann cells.

**Figure 3:**
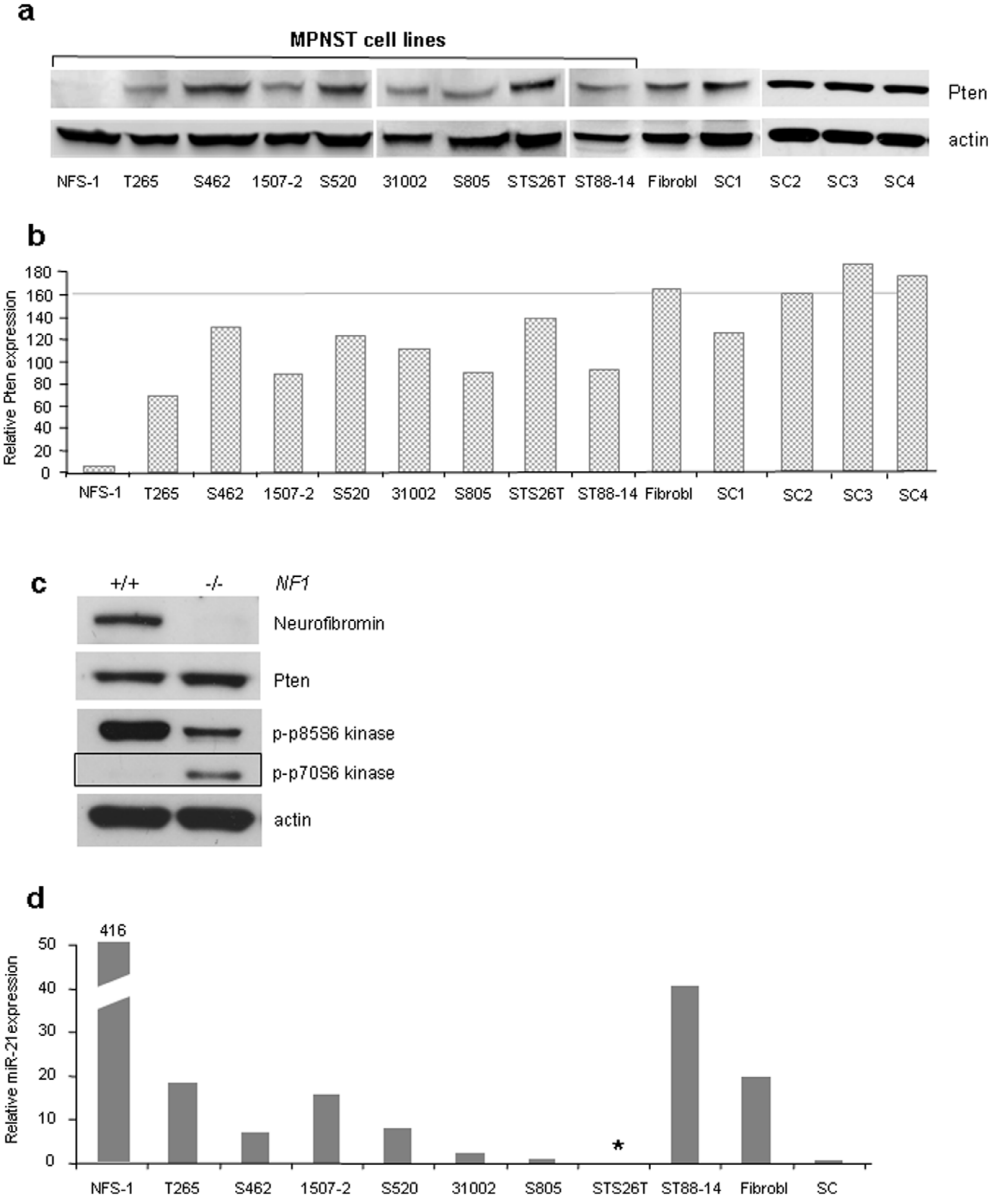
Western blot analysis of Pten and p-p70S6 kinase (isoform p-p70 of the S6 kinase, indicates mTOR activation) and examination of miR-21 expression by real time PCR a) Pten analysis of MPNST cell lines, dermal fibroblasts and neurofibroma derived Schwann cell cultures (SC1-4). The grey line indicates mean Pten expression of 4 Schwann cell cultures. b) Quantification of Pten expression (normalized with β-actin). c) Expression of Pten and p-p70S6 kinase in *NF1* positive (+/+) and *NF1* negative (−/−) Schwann cells. d) Relative expression of miR-21 as determined by real time PCR. * Endogeneous control RNU44 was not detectable. This cell line was thus omitted from analysis. SC = neurofibroma derived Schwann cells.

Next, we determined Pten expression in 9 MPNST cell lines, 4 neurofibroma derived Schwann cell cultures and dermal fibroblasts by western blot (Figure 3a & b). We observed that MPNST cell lines expressed Pten at lower levels than Schwann cell cultures or dermal fibroblasts. The only cell line with undetectable Pten expression was NFS-1.

### Pten is not Regulated by Neurofibromin

Less Pten positive cells were detected in MPNST when compared to neurofibroma (Figure 1a). This finding parallels the situation of neurofibromin positive cells in these tumours. To determine if Pten might possibly be regulated by neurofibromin we examined murine embryonal fibroblasts (MEFs) and human Schwann cells with *NF1+/+*and *NF1*−/− status for Pten expression. Pten protein expression was similar in *NF1+/+*and *NF1*−/− negative cells, however, p-p70S6 kinase, an indicator for mTOR activity, was elevated in case of *NF1* deficiency (Figure 3c). The antibody also recognizes the p85 isoform of the S6 kinase. This isoform is, however, not activated by Akt/mTOR.

In case of MEFs we tested the impact of different culture conditions and also determined activation of the Ras/MAPK pathway. An increased level of p-p70S6 kinase and of p-MAPK was detected in *Nf1*−/− MEFs when they were kept in DMEM without serum or in PBS as shown in Figure S1. In a second round of experiments we determined p-mTOR in MEFs kept in PBS for different times. Usage of the Mesoscale system was more sensitive and allowed better resolution than western blot. We could confirm stronger basal mTOR activity in *Nf1*−/− cells (Figure S1b).

### Mechanisms of *PTEN* Regulation

Since NF1 status had no influence on Pten expression, we proceeded with further experiments to elucidate mechanisms responsible for Pten downregulation in MPNST.

To analyse an involvement of *PTEN* mutations we examined all *PTEN* coding exons in 24 solid MPNST and seven MPNST cell lines. In addition, we screened for *PIK3CA* sequence alterations, since somatic mutations in this gene can also lead to Akt/mTOR pathway activation. Activating mutations of the p110alpha subunit of Pi3k, encoded by the *PIK3CA* gene, have been identified in a broad spectrum of tumours, e.g. breast cancer and glioblastomas. We analysed exon 1, 9 and 20, which have been identified as hot spot regions for mutations [20] in 38 MPNST. Somatic mutations were neither detected in *PTEN* nor in *PIK3CA*.

Next, we determined *PTEN* promotor methylation, a common mechanism for Pten down-regulation. A total of 73 samples (55 solid tumours and 18 cell cultures) was analysed by quantitative positional methylation analysis. We focused on methylation of CpG island 3, which contains 5 CpG sides [13]. Comparison of 29 benign nerve sheath tumours (5 neurinomas, 24 neurofibromas) and 26 solid MPNST (3 sporadic and 23 NF1-associated MPNST) revealed a significantly higher methylation frequency in MPNST (p = 0.001, Pearson correlation). With the exception of 3 neurofibromas, which contained 10%, 23% and 30% methylated DNA, all other benign tumour samples were unmethylated. Five MPNST had DNA methylation levels >50%, two between 30–50% and four between 8–29%. Standard deviation between individual CpG sides was generally small (<10%). Clinical data were available for 21 MPNST patients including 3 sporadic cases without metastasis. The tumours were grouped in two categories: methylation <29% (n = 14) and methylation ≥29% (n = 7). We detected a significant association (log rank p = 0.015) of *PTEN* methylation and appearance of metastasis (Figure S3). Pten immunohistochemistry of the primary MPNST was available for only 14 tumours with clinical data and revealed no significant correlation with metastasis. All MPNST that were analysed with more than one method are listed in Table S1.

Moreover, we analysed 18 cell cultures comprising 9 neurofibroma cultures from NF1 patients, one Schwann cell culture of nervus suralis (non-NF1 patient) and 8 MPNST cell lines. DNA samples from cultures of benign tissues were unmethylated. In contrast, 7 out of 8 MPNST cell lines contained methylated DNA (median 71%, range 12–90%).

RNA of high quality was available from 6 solid MPNST, 9 neurofibromas and several cell cultures (9 MPNST cell lines; *NF1−/−* Schwann cells and dermal fibroblasts). The samples were examined by real-time PCR with a *PTEN* specific minor groove–binding (MGB) probe. *PTEN* transcript expression did not correlate well with *PTEN* promotor methylation and Pten protein levels. Thus, we hypothesized that post transcriptional mechanisms may be involved in Pten regulation. Because microRNAs (miR) are well known regulators of Pten we determined expression of miR-21 and miR-217. miR-21 showed an inverse correlation (p = 0.015; Pearson correlation) with Pten protein levels in MPNST cell lines (Figure 3d). miR-217 was not detectable in neurofibroma derived Schwann cells and the majority of MPNST cell lines (data not shown).

### Pten Expression and Sensitivity to Rapamycin

**Figure 4:**
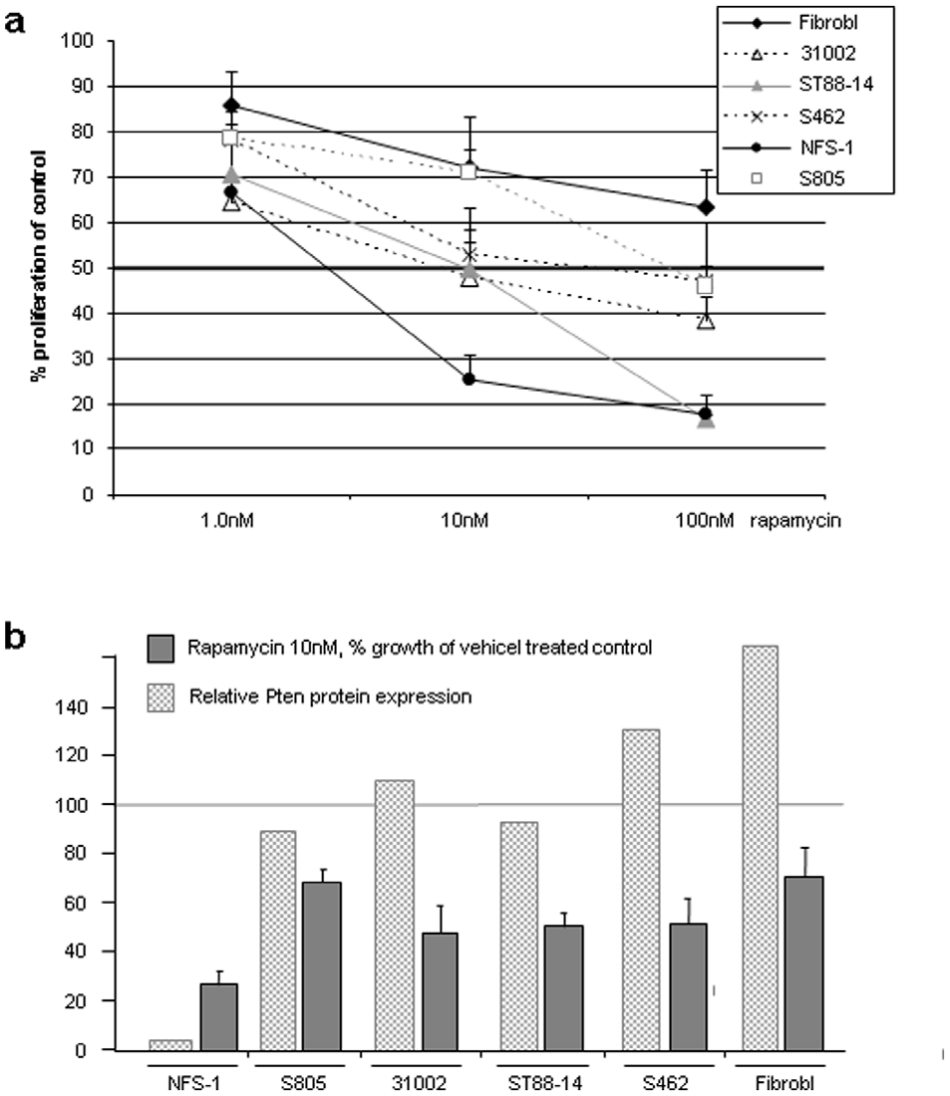
Effect of rapamycin on MPNST cell lines and fibroblasts. a) Dose dependent inhibition of proliferation after 4 days of treatment. b) Pten protein levels correlate with rapamycin sensitivity.

Next, we wanted to know whether the Pten expression status of MPNST cell lines would correlate with their sensitivity to the mTOR inhibitor rapamycin. Using 5 MPNST cell lines and dermal fibroblasts we observed a dose dependant inhibition of proliferation (Figure 4a). The IC50 of rapamycin was around 10 nM for most MPNST cell lines. Highest sensitivity to rapamycin was observed in Pten negative NFS-1 cells (<10 nM) and lowest sensitivity was present in dermal fibroblasts (IC50>100 nM). The correlation of Pten expression and sensitivity to rapamycin was not significant (Figure 4b; p = 0.06; Pearson correlation).

### Synergistic Effect of Rapamycin and Simvastatin

Because statins affect the activity of Ras superfamily members and have been shown to mediate a growth inhibitory effect on MPNST cells we evaluated whether the combination of rapamycin and simvastatin would be synergistic. To find a simvastatin concentration that mediates just a mild anti-proliferative effect as single drug, we tested different concentrations of 0.1 µM, 1.0 µM, and 10 µM. The IC50 of simvastatin on MPNST cell lines ranged between 0.1–1.0 µM. However, a concentration of 10 µM simvastatin was necessary to inhibit fibroblast growth by 50%. Combined treatment approaches with low drug doses were performed (0.1 µM simvastatin and 1.0 nM rapamycin). The results show a synergistic effect of this drug combination in 4 of 5 cell lines (Figure S2a). Because we previously observed an inhibitory effect of the multi-tyrosine kinase inhibitor sunitinib on MPNST cell lines [17] we wanted to analyse sunitinib in combination with rapamycin and simvastatin. A synergistic effect was, however, not achieved when we combined sunitinib with either simvastatin or rapamycin (Figure S2b & c). These combinations actually led to an antagonistic effect in about half of the cell lines.

## Discussion

The aim of this study was to evaluate Pten alterations in human MPNST and neurofibroma and possible consequences for treatment with mTOR inhibitors. We demonstrate that Pten expression in MPNST is significantly lower than in neurofibroma indicating that its down-regulation could contribute to malignant progression.

Because Pten is a key regulator of the Akt/mTOR pathway, the Pten status may impact the therapeutic success of mTOR inhibiting drugs. Previously it was shown that MPNST cell lines are sensitive to mTOR inhibitors in vitro and in vivo [9], [21], [22]. The IC50 of the tested mTOR antagonists (rapamycin and everolimus) was around 10 nM. However, beside NF1 status, the cell lines used in these studies were not examined for other molecular alterations that might cause activation of the Akt/mTOR pathway. We hypothesized that loss of Pten expression could contribute to activation of this pathway and that patients with Pten-deficient tumours might particularly profit from treatment with mTOR inhibitors. In the 5 MPNST cell lines tested no significant correlation between Pten levels and rapamycin sensitivity was detected.

In contrast to solid MPNST, which generally harbour only few Pten positive cells (Fig. 1a) most MPNST cell lines maintain Pten expression although on a lower level compared to Schwann cells (Fig. 3a and b). A possible explanation for this finding is senescence induction by complete loss of *PTEN* expression [24]. Thus, partial Pten expression may have a selective advantage over a complete loss at least in the absence of further mutations able to prevent senescence induction [24].

An impact of Pten status on sensitivity to mTOR inhibitors has been observed in *PTEN-*deficient tumour cells and *Pten-*deficient MEFs, which were both more sensitive to mTOR inhibitors than corresponding Pten positive cells [23]. Another study demonstrated that rapamycin enhanced the sensitivity of Pten*-*deficient glioblastoma cells to treatment with the EGFR inhibitor erlotinib [25]. Moreover, the rapamycin derivate everolimus radiosensitized Pten-deficient prostate cancer cells more than wildtype cells [26]. Our data could not support a clear correlation of Pten expression levels and sensitivity to rapamycin. However, since MPNST are not homogeneous tumours and may harbour different genetic alterations that might influence the Akt/mTOR pathway further studies are needed to clarify the impact of Pten status for therapeutic strategies.

### Combination of Drugs

Although mTOR inhibitors show good effects in vitro, in vivo studies demonstrated their limitation as a single agent. A major problem of targeted therapy is the capability of cancer cells to use alternate pathways for survival and proliferation thereby escaping from specific inhibitors. This problem may be circumvented by either targeting the driving mutation (if there is one) or by drug combinations. Here, we tested different combinations of drugs (Figure S2) and found that the combination of rapamycin and simvastatin yielded best results. Simvastatin, a 3-hydroxy-3-methylglutaryl coenzyme A (HMG CoA) reductase inhibitor, is commonly applied as cholesterol-lowering drug. Anti-tumour effects of statins have been described [27], [28], [29]. The anti-proliferative and pro-apoptotic effects of statins are mediated by multiple mechanisms including blockage of posttranslational modification (prenylation) of Ras superfamily members. Statins have already been shown to improve NF1 symptoms like learning deficits [30], bone healing [31] and to inhibit proliferation of an MPNST cell line [32]. However, simvastatin′s exact mode of action on MPNST cell lines needs further analysis in future studies.

We and others found only a weak additive effect when mTOR inhibitors were combined with receptor tyrosine kinase (RTK) inhibitors (e.g. sunitinib and erlotinib) [21]. Thus, inhibition of signalling molecules downstream of RTKs might be more effective because i) multiple RTKs signal via the same downstream cascades and ii) these pathways may be constitutively activated due to mutations in more than one molecule.

### Significance of Pten in Neural Tissue

Pten is, like neurofibromin, most strongly expressed in the central and peripheral nervous system (PNS). However, the exact cell types expressing Pten in the PNS were not assessed. Here we show that Pten is strongly expressed in axons and Schwann cells. Subtle reduction in Pten expression could predispose to tumourigenesis in a tissue-specific manner. Neoplasms most commonly observed in NF1 patients are nerve sheath tumours, which originate from Schwann cell precursors. It is tempting to speculate that cell types with strong basal Pten expression are particularly in danger of undergoing malignant transformation upon Pten loss or down-regulation. Pten deficiency is frequently observed in primary glioblastomas but not in astrocytomas grade I-III, underlining its role in malignant progression [33]. Since the mTOR pathway is already activated in NF1-associated tumours due to a lack of *NF1*, it is likely that further activation of the Akt/mTOR pathway due to Pten loss promotes tumour progression in this setting. In animal studies, it was shown that Pten loss altered brain development [34], [35], [36] and caused nerve sheath tumour formation [10], [37].

### Pten Regulation

Regulation of Pten expression is complex and may be caused by multiple mechanisms including allelic loss, point mutations, epigenetic silencing, miRs, protein stability (interaction with other proteins) and ubiquitination [24]. Previously, we found reduced *PTEN* gene dosage in more than half of MPNST [7]. Here, we screened for *PTEN* mutations, determined promotor methylation, and the expression of Pten-regulating miRs. Finally, we correlated these data with Pten transcription and protein levels. We can exclude, that *PTEN* mutations play a major role in its regulation or function. Nevertheless, our previous finding of frequent mono-allelic loss in MPNST is likely to account for decreased Pten expression. *PTEN* haploinsufficiency or even subtle Pten down-regulation by 20% has been shown to promote tumour development or progression [38], [39]. It was therefore proposed that loss of Pten may be regarded as a continuum rather than a stepwise process [24].

A gene dosage effect in tumour development is also known from patients with PTEN harmatoma tumour syndrome, which is caused by a mono-allelic *PTEN* germ line mutation. Tumours from these patients do not always carry *PTEN* mutations in the second allele [40].

Moreover, *PTEN* promotor methylation correlated significantly with development of metastasis. However, since clinical data was only available for 21 MPNST and metastasis development is influenced by many different factors our finding needs to be confirmed in a larger set of well characterized patients.

A correlation between promotor methylation and transcription was less clear. A possible explanation may be the small set of tumours for which both, DNA and RNA, was available. Moreover, the effect may be masked by other Pten-regulating mechanisms. Since Pten protein and transcript levels did not correlate well in all tumours additional posttranscriptional mechanisms may play a role in Pten regulation. miRs interfere with transcript translation and have been reported to play an important role in Pten regulation [41]. We observed an inverse correlation between miR-21 and Pten protein levels (Fig. 3b & d). miR-21 is among the most commonly and dramatically upregulated miRs in many cancers and is a well known Pten regulator [41], [42]. Thus, our data point to a possible role of miR-21 in the regulation of Pten regulation in nerve sheath tumours. However, this issue needs further evaluation and should include larger panels of primary tumours. Taken together, our data suggest that a combination of multiple mechanisms leads to altered Pten expression in nerve sheath tumours.

In summary, accumulating evidence supports the assumption of Pten being an important player in MPNST development. Whether determination of Pten expression status in MPNST might assist in refining therapy needs further evaluation in clinical studies. However, we suggest that approaches with mTOR inhibitors in combination with other agents (e.g. conventional chemotherapy or statins) could improve therapeutic success.

## Supporting Information

Figure S1 Lack of *Nf1* does not alter Pten expression but activates the Ras/MAPK and mTOR pathway. a) MEFs of the indicated genotype were kept under different conditions and analysed by western blot. The boxes mark culture conditions, where differences between the two genotypes are most pronounced. b) The assay was repeated with slightly different culture conditions. The significance of *Nf1* status for mTOR activation was determined as the ration between p-mTOR to total mTOR. All samples were tested in duplicates.
(TIF)

Figure S2 Anti-proliferative effect of different drugs on MPNST cell lines and fibroblasts. a) simvastatin, rapamycin and the combination of both drugs b) rapamycin, sunitinib and the combination of both drugs c) simvastatin, sunitinib and the combination of both drugs
(TIF)

Figure S3 Kaplan-Meier curve shows metastasis free time in patients with promotor methylation<29% and ≥29%.
(TIF)

Figure S4 Nuclear Pten localization in MPNST and neurofibroma was determined by immunohistochemistry. Each dot represents one tumour. The difference of nuclear Pten in MPNST and neurofibroma was not significant (p = 0.1, unpaired t-test).
(TIF)

Table S1 Molecular analysis and clinical data of MPNST patients.
(DOCX)

## References

[1] Huson SM (1994) Neurofibromatosis 1: a clinical and genetic overview. In: Huson SM, Hughes RAC, editors. The Neurofibromatoses. London: Chapman and Hall Medical. 160–203.

[2] Evans DG, Baser ME, McGaughran J, Sharif S, Howard E, et al (2002) Malignant peripheral nerve sheath tumours in neurofibromatosis 1. J Med Genet 39: 311–314. 12011145 10.1136/jmg.39.5.311 PMC1735122

[3] Legius E, Dierick H, Wu R, Hall BK, Marynen P, et al (1994) TP53 mutations are frequent in malignant NF1 tumors. Genes Chromosomes Cancer 10: 250–255. 7522538 10.1002/gcc.2870100405

[4] Kourea HP, Orlow I, Scheithauer BW, Cordon-Cardo C, Woodruff JM (1999) Deletions of the INK4A gene occur in malignant peripheral nerve sheath tumors but not in neurofibromas. Am J Pathol 155: 1855–1860. 10595915 10.1016/S0002-9440(10)65504-6 PMC1866948

[5] Perry A, Kunz SN, Fuller CE, Banerjee R, Marley EF, et al (2002) Differential NF1, p16, and EGFR patterns by interphase cytogenetics (FISH) in malignant peripheral nerve sheath tumor (MPNST) and morphologically similar spindle cell neoplasms. J Neuropathol Exp Neurol 61: 702–709. 12152785 10.1093/jnen/61.8.702

[6] Holtkamp N, Okuducu AF, Mucha J, Afanasieva A, Hartmann C, et al (2006) Mutation and expression of PDGFRA and KIT in malignant peripheral nerve sheath tumors, and its implications for imatinib sensitivity. Carcinogenesis 27: 664–671. 16357008 10.1093/carcin/bgi273

[7] Holtkamp N, Malzer E, Zietsch J, Okuducu AF, Mucha J, et al (2008) EGFR and erbB2 in malignant peripheral nerve sheath tumors and implications for targeted therapy. Neuro Oncol 10: 946–957. 18650488 10.1215/15228517-2008-053 PMC2719009

[8] Perrone F, Da Riva L, Orsenigo M, Losa M, Jocolle G, et al (2009) PDGFRA, PDGFRB, EGFR, and downstream signaling activation in malignant peripheral nerve sheath tumor. Neuro Oncol 11: 725–736. 19246520 10.1215/15228517-2009-003 PMC2802393

[9] Johannessen CM, Reczek EE, James MF, Brems H, Legius E, et al (2005) The NF1 tumor suppressor critically regulates TSC2 and mTOR. Proc Natl Acad Sci U S A 102: 8573–8578. 15937108 10.1073/pnas.0503224102 PMC1142482

[10] Gregorian C, Nakashima J, Dry SM, Nghiemphu PL, Smith KB, et al (2009) PTEN dosage is essential for neurofibroma development and malignant transformation. Proc Natl Acad Sci U S A 106: 19479–19484. 19846776 10.1073/pnas.0910398106 PMC2765459

[11] Hartmann C, Devermann L, Gehlhaar C, Holtkamp N, von Deimling A (2006) PIK3CA mutations in oligodendroglial tumours. Neuropathol Appl Neurobiol 32: 209–212. 16599949 10.1111/j.1365-2990.2006.00700.x

[12] Hartmann C, Nuemann A, Mueller W, Holtkamp N, Simon M, et al (2004) Fine mapping of chromosome 22q tumor suppressor gene candidate regions in astrocytoma. Int J Cancer 108: 839–844. 14712485 10.1002/ijc.11638

[13] Mirmohammadsadegh A, Marini A, Nambiar S, Hassan M, Tannapfel A, et al (2006) Epigenetic silencing of the PTEN gene in melanoma. Cancer Res 66: 6546–6552. 16818626 10.1158/0008-5472.CAN-06-0384

[14] Frahm S, Mautner VF, Brems H, Legius E, Debiec-Rychter M, et al (2004) Genetic and phenotypic characterization of tumor cells derived from malignant peripheral nerve sheath tumors of neurofibromatosis type 1 patients. Neurobiol Dis 16: 85–91. 15207265 10.1016/j.nbd.2004.01.006

[15] Dahlberg WK, Little JB, Fletcher JA, Suit HD, Okunieff P (1993) Radiosensitivity in vitro of human soft tissue sarcoma cell lines and skin fibroblasts derived from the same patients. Int J Radiat Biol 63: 191–198. 8094415 10.1080/09553009314550251

[16] Glover TW, Stein CK, Legius E, Andersen LB, Brereton A, et al (1991) Molecular and Cytogenetic analysis of tumors in von Recklinghausen neurofibromatosis. Genes Chrom Cancer 3: 62–70. 1906341 10.1002/gcc.2870030111

[17] Zietsch J, Ziegenhagen N, Heppner FL, Reuss D, von Deimling A, et al (2010) The 4q12 amplicon in malignant peripheral nerve sheath tumors: consequences on gene expression and implications for sunitinib treatment. PLoS One 5: e11858. 20686603 10.1371/journal.pone.0011858 PMC2912277

[18] Jacks T, Shih TS, Schmitt EM, Bronson RT, Bernards A, et al (1994) Tumour predisposition in mice heterozygous for a targeted mutation in Nf1. Nat Genet 7: 353–361. 7920653 10.1038/ng0794-353

[19] Serra E, Rosenbaum T, Winner U, Aledo R, Ars E, et al (2000) Schwann cells harbor the somatic NF1 mutation in neurofibromas: evidence of two different Schwann cell subpopulations. Hum Mol Genet 9: 3055–3064. 11115850 10.1093/hmg/9.20.3055

[20] Samuels Y, Wang Z, Bardelli A, Silliman N, Ptak J, et al (2004) High frequency of mutations of the PIK3CA gene in human cancers. Science 304: 554. 15016963 10.1126/science.1096502

[21] Johansson G, Mahller YY, Collins MH, Kim MO, Nobukuni T, et al (2008) Effective in vivo targeting of the mammalian target of rapamycin pathway in malignant peripheral nerve sheath tumors. Mol Cancer Ther 7: 1237–1245. 18483311 10.1158/1535-7163.MCT-07-2335 PMC2855168

[22] Bhola P, Banerjee S, Mukherjee J, Balasubramanium A, Arun V, et al (2009) Preclinical in vivo evaluation of rapamycin in human malignant peripheral nerve sheath explant xenograft. Int J Cancer 126: 563–571. 10.1002/ijc.24783 19634141

[23] Neshat MS, Mellinghoff IK, Tran C, Stiles B, Thomas G, et al (2001) Enhanced sensitivity of PTEN-deficient tumors to inhibition of FRAP/mTOR. Proc Natl Acad Sci U S A 98: 10314–10319. 11504908 10.1073/pnas.171076798 PMC56958

[24] Salmena L, Carracedo A, Pandolfi PP (2008) Tenets of PTEN tumor suppression. Cell 133: 403–414. 18455982 10.1016/j.cell.2008.04.013

[25] Wang MY, Lu KV, Zhu S, Dia EQ, Vivanco I, et al (2006) Mammalian Target of Rapamycin Inhibition Promotes Response to Epidermal Growth Factor Receptor Kinase Inhibitors in PTEN-Deficient and PTEN-Intact Glioblastoma Cells. Cancer Res 66: 7864–7869. 16912159 10.1158/0008-5472.CAN-04-4392

[26] Cao C, Subhawong T, Albert JM, Kim KW, Geng L, et al (2006) Inhibition of mammalian target of rapamycin or apoptotic pathway induces autophagy and radiosensitizes PTEN null prostate cancer cells. Cancer Res 66: 10040–10047. 17047067 10.1158/0008-5472.CAN-06-0802

[27] Mo H, Elson CE (2004) Studies of the isoprenoid-mediated inhibition of mevalonate synthesis applied to cancer chemotherapy and chemoprevention. Exp Biol Med (Maywood) 229: 567–585. 15229351 10.1177/153537020422900701

[28] Graaf MR, Richel DJ, van Noorden CJ, Guchelaar HJ (2004) Effects of statins and farnesyltransferase inhibitors on the development and progression of cancer. Cancer Treat Rev 30: 609–641. 15531395 10.1016/j.ctrv.2004.06.010

[29] Khanzada UK, Pardo OE, Meier C, Downward J, Seckl MJ, et al (2006) Potent inhibition of small-cell lung cancer cell growth by simvastatin reveals selective functions of Ras isoforms in growth factor signalling. Oncogene 25: 877–887. 16170339 10.1038/sj.onc.1209117

[30] Li W, Cui Y, Kushner SA, Brown RA, Jentsch JD, et al (2005) The HMG-CoA reductase inhibitor lovastatin reverses the learning and attention deficits in a mouse model of neurofibromatosis type 1. Curr Biol 15: 1961–1967. 16271875 10.1016/j.cub.2005.09.043

[31] Kolanczyk M, Kuhnisch J, Kossler N, Osswald M, Stumpp S, et al (2008) Modelling neurofibromatosis type 1 tibial dysplasia and its treatment with lovastatin. BMC Med 6: 21. 18671844 10.1186/1741-7015-6-21 PMC2516519

[32] Sane KM, Mynderse M, Lalonde DT, Dean IS, Wojtkowiak JW, et al (2010) A novel geranylgeranyl transferase inhibitor in combination with lovastatin inhibits proliferation and induces autophagy in STS-26T MPNST cells. J Pharmacol Exp Ther 333: 23–33. 20086055 10.1124/jpet.109.160192 PMC2846025

[33] Reifenberger G, Collins VP (2004) Pathology and molecular genetics of astrocytic gliomas. J Mol Med 82: 656–670. 15316624 10.1007/s00109-004-0564-x

[34] Backman SA, Stambolic V, Suzuki A, Haight J, Elia A, et al (2001) Deletion of Pten in mouse brain causes seizures, ataxia and defects in soma size resembling Lhermitte-Duclos disease. Nat Genet 29: 396–403. 11726926 10.1038/ng782

[35] Groszer M, Erickson R, Scripture-Adams DD, Lesche R, Trumpp A, et al (2001) Negative regulation of neural stem/progenitor cell proliferation by the Pten tumor suppressor gene in vivo. Science 294: 2186–2189. 11691952 10.1126/science.1065518

[36] Kwon CH, Zhu X, Zhang J, Knoop LL, Tharp R, et al (2001) Pten regulates neuronal soma size: a mouse model of Lhermitte-Duclos disease. Nat Genet 29: 404–411. 11726927 10.1038/ng781

[37] Mawrin C, Kirches E, Boltze C, Dietzmann K, Roessner A, et al (2002) Immunohistochemical and molecular analysis of p53, RB, and PTEN in malignant peripheral nerve sheath tumors. Virchows Arch 440: 610–615. 12070601 10.1007/s00428-001-0550-4

[38] Kwabi-Addo B, Giri D, Schmidt K, Podsypanina K, Parsons R, et al (2001) Haploinsufficiency of the Pten tumor suppressor gene promotes prostate cancer progression. Proc Natl Acad Sci U S A 98: 11563–11568. 11553783 10.1073/pnas.201167798 PMC58769

[39] Alimonti A, Carracedo A, Clohessy JG, Trotman LC, Nardella C, et al (2010) Subtle variations in Pten dose determine cancer susceptibility. Nat Genet 42: 454–458. 20400965 10.1038/ng.556 PMC3118559

[40] Marsh DJ, Coulon V, Lunetta KL, Rocca-Serra P, Dahia PL, et al (1998) Mutation spectrum and genotype-phenotype analyses in Cowden disease and Bannayan-Zonana syndrome, two hamartoma syndromes with germline PTEN mutation. Hum Mol Genet 7: 507–515. 9467011 10.1093/hmg/7.3.507

[41] Meng F, Henson R, Wehbe-Janek H, Ghoshal K, Jacob ST, et al (2007) MicroRNA-21 regulates expression of the PTEN tumor suppressor gene in human hepatocellular cancer. Gastroenterology 133: 647–658. 17681183 10.1053/j.gastro.2007.05.022 PMC4285346

[42] Volinia S, Calin GA, Liu CG, Ambs S, Cimmino A, et al (2006) A microRNA expression signature of human solid tumors defines cancer gene targets. Proc Natl Acad Sci U S A 103: 2257–2261. 16461460 10.1073/pnas.0510565103 PMC1413718

