# Supplementary material for: Impaired Pten Expression in Human Malignant Peripheral Nerve Sheath Tumours
Source: PLoS One. 2012 Nov 6;7(11):e47595. doi: 10.1371/journal.pone.0047595 (PMC3490977; doi:10.1371/journal.pone.0047595)
Supplement: Table S1 — Molecular analysis and clinical data of MPNST patients. (DOCX) [file pone.0047595.s005.docx]

Table S1: Molecular analysis and clinical data of MPNST patients

| Tumor ID | *PTEN* qPCR | *PTEN* Methylation | Pten IHC | *PTEN* MLPA | *PTEN* Mut | *PIK3CA* Mut | Follow up month |
| --- | --- | --- | --- | --- | --- | --- | --- |
| 168 | NA | 1 | NA | n | wt | wt | NA |
| 524 | NA | 0 | NA | R | wt | wt | NA |
| 5050 | NA | 0 | NA | n | wt | wt | NA |
| 21852 | 2050 | 1 | 1% | R | wt | wt | 24 |
| 21914 | 1172 | 0 | 15% | R | wt | wt | 30 |
| 24256 | NA | 3 | NA | R | wt | wt | **132** |
| 24308 | NA | 0 | NA | R | wt | wt | **6** |
| 24324 | NA | 3 | NA | n | wt | NA | 46 |
| 24472 | 1498 | 1 | 5% | n | wt | wt | 11 |
| 24476 | NA | 0 | NA | NA | wt | wt | 99 |
| 24480 | NA | 1 | 5% | R | wt | NA | 7 |
| 24484 | NA | 0 | NA | n | wt | wt | 18 |
| 24668 | NA | 3 | NA | NA | wt | wt | **0** |
| 24670 | NA | 3 | NA | R | wt | wt | **4** |
| 24694 | NA | 0 | 10% | R | wt | wt | 29 |
| 26582***** | 231 | 0 | 0% | R | wt | wt | 126 |
| 26584***** | 2588 | 2 | 5% | Amp/R | wt | wt | 47 |
| 26588***** | NA | 0 | 30% | R | wt | wt | 63 |
| 26592 | 2704 | 0 | 5% | NA | wt | wt | 0 |
| 27724***** | NA | 0 | 5% | NA | wt | wt | **7** |
| 28650***** | NA | 0 | 70% | n | NA | wt | 12 |
| 28652***** | NA | 0 | 60% | n | wt | wt | 15 |
| 29250 | NA | 0 | 5% | n | wt | NA | NA |
| 31472 | NA | 3 | 5% | 1.4amp | wt | NA | **22** |
| 31474 | NA | 2 | 1% | n | wt | NA | 4 |

| Cell lines | *PTEN* qPCR | *PTEN* Methylation | Pten WB | *PTEN* MLPA | *PTEN* Mut | *PIK3CA* Mut |
| --- | --- | --- | --- | --- | --- | --- |
| NSF-1 | 52 | 0 | 9 | R | wt | NA |
| T265 | 1 | 3 | 69 | NA | wt | NA |
| S462 | 120 | 1 | 131 | N | wt | NA |
| 1507-2 | 0 | 3 | 88 | NA | NA | NA |
| S520 | 22 | 3 | 122 | NA | wt | NA |
| 31002***** | 235 | 1 | 110 | R | wt | NA |
| S805 | 11 | 3 | 90 | NA | wt | NA |
| STS26T***** | NA | NA | 138 | NA | wt | NA |
| ST88-14 | 18 | 3 | 93 | R | wt | NA |

The table lists all solid MPNST (upper part) and MPNST cell lines (lower part) for which more than one analysis is available. ID: tumor identification number. Transcript levels were quantified by real time PCR (*PTEN* qPCR) and are given as relative expression. *PTEN* methylation: 0≤8%; 1=8-29%; 2=30-50%; 3= >50%. Pten protein was quantified in the primary MPNST by immunohistochemistry (Pten IHC: positive cells/HPF) or in case of cell lines by western blot by densitometric analysis (Pten WB). Mutation analysis (Mut) is provided for *PTEN* and *PIK3CA*. Clinical follow up in month is provided (Follow up month). Patients with metastasis are printed in bold. *PTEN* gene dosage analysis was performed by MLPA: R indicates mono-allelic loss, L indicates bi-allelic loss, n corresponds to normal gene dosage and Amp to increased gene dosage. Sporadic MPNST are marked with an asterisk. NA: not assessed. *PTEN* MLPA data have been published previously [[7](#_ENREF_7)].
